# The Last Fashion with Medical Journalists

**Published:** 1884-09

**Authors:** 


### THE LAST FASHION WITH MEDICAL JOURNALISTS.

Under this head the editor of *Gaillard's Medical Journal* vents his spleen upon such journals as dare to mention an advertiser of any kind, in the following words:

"If these writers and so-called editors are not ashamed of themselves, their friends are at least ashamed of them. This selling of their editorial columns, directly and indirectly, is a shame on respectable journalism. It is remarkable that such men take their snbscribers to be idiots, in supposing that they can be deceived by such editorial twaddle, venalism, and mendacity."

What does the editor of *Gaillard's Journal* say of an editor who reviews a book that is nothing more than an advertisement, and who does not refer at all to the fact that it is an advertisement, when it is so characterized by others? For our part we can see no harm in giving responsible advertisers a notice when you have that notice properly displayed, that readers may not be misled; and feeling that way, we shall continue to give advertisers such notices as they deserve.

